# First report of molecular genotyping, pathotyping, and histopathological characterization of lentogenic genotype II Newcastle disease virus circulating in young ostrich flocks in Egypt

**DOI:** 10.14202/vetworld.2026.2951-2970

**Published:** 2026-07-14

**Authors:** Eman Abd-El Monum Shosha, Ibrahim Eldaghayes, Ali Mahmoud Zanaty, Rania M. Elbatawy, Sara Abdelnaser, Ahmed Fotouh

**Affiliations:** 1 Virology Department, Faculty of Veterinary Medicine, New Valley University, El-Khargia 72511, Egypt.; 2 Department of Microbiology and Parasitology, Faculty of Veterinary Medicine, University of Tripoli, Tripoli, Libya.; 3 Gene Analysis Unit, Reference Laboratory for Quality Control on Poultry (Poultry Diseases Specialty), Animal Health Institute, Agriculture Research Center, Giza 12619, Egypt.; 4 Pathology Department, Faculty of Veterinary Medicine, Moshtohor, Benha University, Egypt.; 5 Pathology and Clinical Pathology Department, Faculty of Veterinary Medicine, New Valley University, El-Khargia, Egypt.

**Keywords:** biosecurity, Egypt, genotype II, molecular epidemiology, Newcastle disease virus, ostrich, pathotyping, phylogenetic analysis

## Abstract

**Background and Aim:**

Newcastle disease virus (NDV) is one of the most economically important avian pathogens worldwide, yet information regarding its molecular epidemiology and pathological characteristics in ostriches remains limited. This study investigated the prevalence, molecular characteristics, pathotype, phylogenetic relationships, and histopathological changes associated with NDV circulating in young ostrich flocks in Egypt.

**Materials and Methods:**

A total of 60 tissue samples were collected from diseased, non-vaccinated ostriches aged 3 weeks to 2 months from eight farms across four Egyptian governorates during 2024. Virus isolation was performed using specific-pathogen-free embryonated chicken eggs, followed by hemagglutination and hemagglutination inhibition assays. Pathogenicity was determined using the mean death time (MDT) and intracerebral pathogenicity index (ICPI). Molecular detection was conducted by real-time reverse-transcription polymerase chain reaction targeting the matrix gene, followed by partial fusion gene amplification, sequencing, and phylogenetic analysis. Histopathological examination was performed on major organs, and positive samples were additionally screened for avian influenza virus, infectious bronchitis virus, and infectious bursal disease virus to exclude coinfections.

**Results:**

Eight of 60 samples tested positive for NDV, with the highest prevalence detected in Ismailia governorate. All positive samples were negative for the tested coinfecting viruses. The isolates exhibited a hemagglutination titer of 9 log₂ hemagglutination units/mL and a hemagglutination inhibition titer of 6 log₂. Biological pathotyping confirmed a lentogenic pathotype with an MDT of 96 h and an ICPI of 0.4. Phylogenetic analysis classified the isolates within genotype II, class II, possessing the characteristic lentogenic fusion protein cleavage motif ¹¹²GRQGRL¹¹⁷. The isolates shared 97%–99% nucleotide identity with commonly used vaccine strains, including LaSota, Hitchner, and Clone 30. Histopathological examination revealed marked lesions in the respiratory and digestive systems, including epithelial degeneration, hemorrhage, inflammatory infiltrates, lymphoid depletion, hepatic necrosis, and intestinal villous damage, despite the lentogenic nature of the virus.

**Conclusion:**

This study provides the first comprehensive molecular, pathotyping, phylogenetic, and histopathological characterization of lentogenic genotype II NDV circulating in young ostriches in Egypt. The findings demonstrate that lentogenic vaccine-related genotype II strains can induce clinically relevant pathological alterations in ostriches, emphasizing the need for continuous molecular surveillance, host-specific vaccination strategies, enhanced biosecurity, and integrated monitoring of ostriches, poultry, and wild birds to reduce NDV transmission and evolution.

## INTRODUCTION

Viral diseases continue to pose a serious threat to animal health and poultry production worldwide, causing recurrent outbreaks with substantial economic losses and adverse impacts on food security [1–5]. Most of these outbreaks are caused by RNA viruses, which are characterized by high mutation rates and rapid evolution, facilitating viral adaptation, transmission, and persistence, particularly in backyard poultry production systems [6–9]. Among these pathogens, Newcastle disease virus (NDV) remains one of the most economically important avian viruses because of its high transmissibility, rapid spread, elevated mortality, impaired growth performance, and reduced egg production [10]. Previously known as avian paramyxovirus-1, NDV is currently classified by the International Committee on Taxonomy of Viruses as *Avian orthoavulavirus 1*, belonging to the genus *Orthoavulavirus*, subfamily *Paramyxovirinae*, and family *Paramyxoviridae* [11]. NDV possesses an enveloped, single-stranded, negative-sense, non-segmented RNA genome of approximately 15 kilobases encoding eight viral proteins, including the two major surface glycoproteins, fusion (F) and hemagglutinin-neuraminidase (HN) [12, 13]. The hemagglutinin-neuraminidase glycoprotein mediates viral attachment to host cells, whereas the F protein facilitates membrane fusion, viral entry, and hemolytic activity [13–16].

The clinical severity of NDV infection varies considerably depending on viral strain, concurrent infections, immune status, bird age, environmental conditions, and host species, with mortality rates reaching 100% during outbreaks caused by highly virulent strains [17, 18]. Based on pathogenicity and clinical manifestations, NDV strains are classified into four pathotypes: lentogenic strains, which generally cause subclinical or mild respiratory disease and are widely used as vaccine strains; mesogenic strains, which produce moderate respiratory disease, decreased egg production, and occasional neurologic signs; velogenic strains, which cause severe systemic disease associated with high mortality; and asymptomatic enteric strains, which produce inapparent intestinal infections [19, 20]. In Egypt, NDV control primarily depends on vaccination programs and strict biosecurity measures using live, inactivated, and genetically modified vaccines, predominantly derived from genotypes I and II. However, the continuous genetic evolution of NDV compromises vaccine efficacy and remains a major challenge for disease control [19, 20].

NDV virulence is primarily determined by the amino acid composition of the F protein cleavage site, where proteolytic activation of the precursor F protein is essential for viral infectivity [21]. Velogenic strains possess multiple basic amino acids at the cleavage site, allowing cleavage by ubiquitous intracellular proteases and facilitating systemic dissemination. In contrast, lentogenic strains contain monobasic cleavage motifs that restrict viral replication largely to the respiratory and intestinal mucosa [21]. Based on *F* gene sequencing and phylogenetic analysis, NDV isolates are classified into two major classes despite belonging to a single serotype. Class I comprises predominantly nonvirulent viruses circulating in wild aquatic birds, whereas class II includes most virulent viruses infecting domestic poultry and is currently divided into twenty-one recognized genotypes (I–XXI) [10, 20]. Since the late 1980s, genotype VII has been the predominant lineage responsible for the fourth global NDV pandemic and remains the principal genotype associated with outbreaks in Egypt despite intensive vaccination programs [22–24]. Nevertheless, genotypes II and VI continue to circulate in Egypt and other parts of North Africa, highlighting the dynamic molecular epidemiology of NDV in the region [25].

NDV has a remarkably broad host range and infects more than 250 avian species, including ostriches (*Struthio camelus*) [26]. In Egypt, ostrich farming has expanded due to its commercial value; however, the industry continues to incur substantial production losses from low hatchability, embryonic mortality exceeding 30%, poor chick survival, and infectious diseases [27, 28]. NDV was first reported in Egyptian ostrich farms in 2010, where outbreaks were associated with high mortality and neurologic manifestations in susceptible birds. Infection in ostriches represents an important veterinary and economic concern because it reduces productivity, facilitates viral maintenance within mixed-species production systems, and may contribute to disease transmission among ostriches, commercial poultry, and wild birds, thereby posing additional biosecurity challenges [28–30].

Although NDV has been extensively investigated in chickens and other domestic poultry, information regarding its molecular epidemiology, biological characteristics, and pathological manifestations in ostriches remains scarce. Published studies have largely focused on outbreak descriptions or on the detection of virulent NDV strains, whereas comprehensive investigations that integrate virus isolation, biological pathotyping, molecular detection, phylogenetic characterization, and histopathological evaluation of naturally infected ostriches are lacking. Furthermore, the circulating genotypes, molecular pathotypes, and tissue lesions associated with naturally occurring lentogenic genotype II NDV in Egyptian ostriches remain poorly understood. This knowledge gap limits understanding of host-specific disease expression, the epidemiological role of ostriches in NDV maintenance and transmission, and the implications of vaccine-related genotype II viruses for surveillance and control strategies in Egypt.

Therefore, this study aimed to comprehensively characterize NDV circulating in naturally infected young ostrich flocks in Egypt through virus isolation, biological pathotyping, molecular detection by real-time reverse-transcription polymerase chain reaction (rRT-PCR), partial *F* gene sequencing, phylogenetic analysis, and histopathological examination. The study further sought to determine the prevalence, genotype, pathotype, and phylogenetic relationships of circulating NDV isolates, evaluate the associated pathological alterations in major organs, and investigate the presence of coinfections with other economically important avian viruses. To the best of our knowledge, this study represents the first comprehensive molecular, biological, phylogenetic, and histopathological characterization of naturally circulating lentogenic genotype II NDV in young Egyptian ostriches, providing valuable baseline information for molecular surveillance, host-specific vaccination strategies, and improved biosecurity programs for the ostrich industry in Egypt.

## MATERIALS AND METHODS

### Ethical approval

The study was conducted in accordance with institutional guidelines for animal welfare and experimental procedures. Ethical approval was obtained from the Institutional Animal Care and Use Committee of the Faculty of Veterinary Medicine, New Valley University, Egypt (Approval No. 07-2025-24). Permission to collect samples was obtained from the owners of all participating ostrich farms before sample collection. All procedures were conducted and reported in accordance with the Animal Research: Reporting of *In Vivo* Experiments (ARRIVE) 2.0 guidelines.

### Study period and location

**Figure 1:**
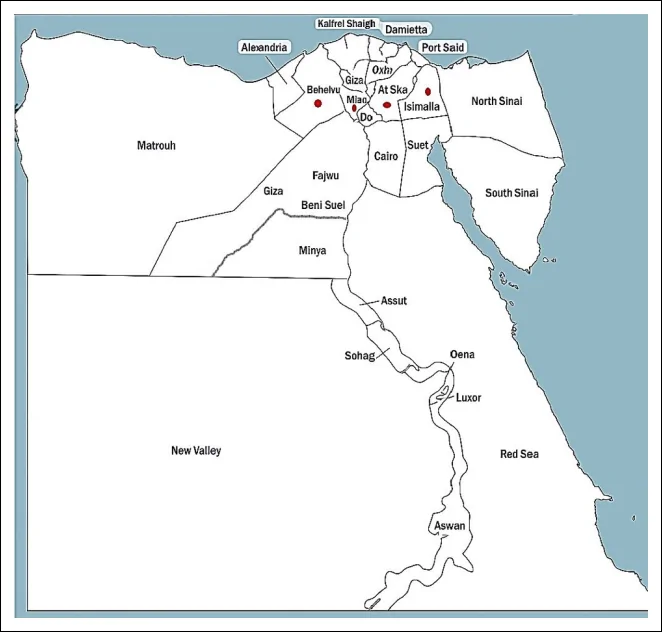
Distribution of Newcastle disease virus-positive ostrich farms in the Egyptian governorates of Ismailia, El-Menofia, El-Behera, and El-Sharquia, represented by red circles.

The study was conducted from January to December 2024. Samples were collected from eight diseased ostrich farms located in four Egyptian governorates: Ismailia, El-Menofia, El-Behera, and El-Sharquia (Figure 1, Table 1).

### Study design

A cross-sectional field investigation was conducted to determine the occurrence and molecular characteristics of NDV circulating in naturally infected young ostriches. The study included clinical assessment, sample collection, virus isolation, molecular detection, biological pathotyping, phylogenetic analysis, and histopathological examination of suspected NDV cases.

### Sample collection and processing

A total of 60 tissue samples were collected from eight ostrich farms with a history of mild respiratory disease characterized by depression, ocular discharge, and mild eyelid edema. The flock size ranged from 50 to 800 birds per farm. None of the investigated flocks had been vaccinated against NDV. Tissue specimens, including the trachea, lungs, intestine, kidney, brain, liver, spleen, and proventriculus, were collected from ostriches aged 3 weeks to 2 months and stored at −80°C until virus isolation and PCR-based molecular detection.

All samples were collected from freshly dead ostriches that died naturally during disease outbreaks; therefore, no euthanasia or experimental sacrifice was performed. Farms were selected based on the presence of clinical signs compatible with NDV infection and recent mortality among young ostriches. Only non-vaccinated flocks with owner consent were included in the study. Freshly dead birds aged 3 weeks to 2 months that died during active outbreaks were selected for tissue collection, whereas severely decomposed carcasses and farms lacking sufficient epidemiological information were excluded.

**The collected tissues were homogenized in sterile phosphate-buffered saline (PBS:** pH 7.2) to prepare a 10% (w/v) tissue suspension supplemented with an antibiotic mixture containing penicillin (1000 IU/mL), streptomycin (2 mg/mL), and gentamicin (2 mg/mL) (Sigma Chemical Company, St. Louis, MO, USA). Following overnight incubation at 4°C, the homogenates were centrifuged at 3000 rpm for 10 minutes, and the clarified supernatants were collected for subsequent virus isolation.

### Virus isolation and hemagglutination assay

Individual tissue homogenates (0.2 mL) were inoculated into the allantoic cavity of 9–11-day-old specific-pathogen-free (SPF)-embryonated chicken eggs (ECE) obtained from the Nile SPF Farm (Koom Oshiem, Fayoum, Egypt). The inoculated eggs were incubated at 37°C for 5 days and monitored daily by candling to evaluate embryo viability [38]. Three successive passages were performed for virus isolation. Embryos that died within the first 24 h post-inoculation were considered to have died from nonspecific causes and were excluded from further analysis. The allantoic fluid was harvested from the remaining embryos and tested for hemagglutination (HA) activity using freshly prepared 1% chicken RBCs.

HA-positive allantoic fluids were subsequently confirmed by hemagglutination inhibition (HI) testing with NDV reference antiserum, following previously described procedures [38]. The LaSota strain (inactivated NDV antigen) was included as a positive control. Both the HA and hemagglutination inhibition (HI) assays were performed according to the procedures described in the World Organization for Animal Health (WOAH) Manual of Diagnostic Tests and Vaccines for Terrestrial Animals, Chapter: Newcastle disease (infection with *Avian orthoavulavirus 1*).

**Table 1:** Descriptive characteristics of ostrich farms investigated for Newcastle disease virus in four Egyptian governorates.

**Flock location**	**Ostrich farms**	**Samples (n)**	**Flock age**	**No. of birds**	**Positive farms**	**Prevalence (%)**	**Trachea**	**Brain**	**Lung**	**Proventriculus**	**Intestine**	**Liver**	**Spleen**	**Kidney**
Ismailia	4	29	4–6 weeks	50–800	4	13.7	4	3	3	8	5	2	1	1
El-Menofia	2	12	3–5 weeks	100–600	2	16.6	2	1	2	4	2	1	1	1
El-Behera	1	9	4–7 weeks	110–550	1	11.1	1	1	2	1	2	–	1	1
El-Sharquia	1	10	4–8 weeks	140–700	1	10.0	1	1	2	3	2	1	-	-

### Biological pathotyping

Biological pathotyping of the NDV isolates was performed by determining the mean death time (MDT) in SPF-ECE and the ICPI in 1-day-old SPF chicks according to established protocols [31]. The SPF-ECE and chicks were obtained from the Nile SPF Farm (Koom Oshiem, Fayoum, Egypt).

For the MDT assay, five 9–11-day-old SPF-ECE were inoculated with each virus dilution according to WOAH recommendations. The ICPI assay was performed by intracerebral inoculation of ten 1-day-old SPF chicks, which were observed daily for 8 consecutive days. All assays were conducted in duplicate.

Sterile PBS-inoculated SPF-ECE and SPF chicks served as negative controls and remained free of clinical signs, lesions, and mortality throughout the experimental period. MDT and ICPI values were interpreted according to the standard WOAH criteria for NDV pathotype classification. Because MDT and ICPI are standardized biological pathotyping assays, the results were interpreted descriptively without additional statistical analysis.

### RNA extraction and rRT-PCR

Viral RNA was extracted from infected allantoic fluid obtained from ten NDV isolates using the QIAamp Viral RNA Mini Kit (Qiagen, Hilden, Germany) according to the manufacturer's instructions. The purity and concentration of the extracted RNA were determined spectrophotometrically prior to amplification to ensure RNA quality for downstream molecular analyses.

Detection of NDV was performed by rRT-PCR targeting the *M* gene using the QuantiTect® Probe RT-PCR Kit (Qiagen) according to the manufacturer's instructions. The primer and probe sequences used for amplification are presented in Table 2 [32]. Samples with cycle threshold values ≤35 were considered positive for NDV RNA. The rRT-PCR assay was performed according to previously validated protocols and the manufacturer's recommendations.

**Table 2:** Primers and probe sets used for Newcastle disease virus detection and sequencing.

**Assay type**	**Primer name**	**Sequence (5′–3′)**	**Target gene**	**Amplicon size**	**Thermal cycling conditions**
rRT-PCR	NDV M-F	AGTGATGTGCTCGGACCTTC	*M *gene	121 bp	Reverse-transcription at 50°C for 30 min, followed by initial activation/denaturation at 95°C for 15 min. Amplification was performed for 40 cycles consisting of denaturation at 94°C for 15 s and annealing/extension at 60°C for 60 s.
rRT-PCR	NDV M-R	CCTGAGGAGAGGCATTTGCTA			
rRT-PCR	NDV M-Probe	[FAM]TTCTCTAGCAGTGGGACAGCCTGC[TAMRA]			
RT-PCR	M2 (Forward)	TGGAGCCAAACCCGCACCTGCGG	*F* gene	766 bp	Reverse-transcription at 50°C for 30 min, followed by preliminary denaturation at 95°C for 15 min. PCR amplification consisted of 35 cycles of denaturation at 94°C for 30 s, annealing at 55°C for 30 s, and extension at 72°C for 1 min, followed by a final extension at 72°C for 10 min.
RT-PCR	F2 (Reverse)	GGAGGATGTTGGCAGCATT			

### Screening for coinfection by RT-qPCR

All NDV-positive samples were subsequently screened for coinfection with Avian influenza virus (AIV), Infectious bronchitis virus (IBV), and Infectious bursal disease virus (IBDV) using specific RT-qPCR assays. The primer and probe sequences used for consensus detection and subtype identification are presented in Table 3 [33–38].

### *F* gene amplification, sequencing, and phylogenetic analysis

NDV-positive samples identified by RT-qPCR were subjected to partial amplification of the *F* gene using the QIAGEN OneStep RT-PCR Kit (Qiagen, Hilden, Germany) according to the manufacturer's instructions. Amplification was performed using the primer pair M2 (forward: 5′-TGGAGCCAAACCCGCACCTGCGG-3′; nucleotides 980–1003 of the *M* gene) and F2 (reverse: 5′-GGAGGATGTTGGCAGCATT-3′; nucleotides 503–485 of the *F* gene), generating a 766-bp amplicon as previously described [39].

The amplified PCR products were separated by electrophoresis on 1.5% agarose gels. The target bands were excised and purified using the QIAquick Gel Extraction Kit (Qiagen) according to the manufacturer's protocol. Purified PCR products were sequenced in both directions using the BigDye™ Terminator v3.1 Cycle Sequencing Kit (Applied Biosystems, Waltham, MA, USA) on an ABI 3500xL Genetic Analyzer (Life Technologies, Carlsbad, CA, USA).

The nucleotide sequence of the amplified *F* gene was deposited in GenBank under accession number PX088918. Nucleotide and deduced amino acid sequences were assembled and aligned using the ClustalW algorithm and compared with published NDV vaccine strains and representative reference strains belonging to classes I and II available in GenBank [40].

Phylogenetic analysis was performed using the maximum-likelihood method with 1000 bootstrap replicates in MEGA version 7.0, applying the Kimura two-parameter substitution model [41]. Raw forward and reverse chromatograms were manually inspected to verify sequence quality and ambiguous base calls before consensus sequence assembly. Low-quality terminal regions were trimmed before generating the final consensus sequences using BioEdit software. Final phylogenetic tree visualization and annotation were performed using MEGA version 7.0 and BioEdit.

Recombination analysis was conducted to investigate the presence of potential recombinant NDV strains using RDP5 software (version 4.97) with multiple detection algorithms, including RDP, BootScan, MaxChi, GENECONV, SiScan, Chimaera, LARD, PhylPro, and 3Seq [42, 43]. A recombination event was considered positive only when supported by at least four independent detection methods.

**Table 3:** Primers and probe sets used for consensus detection and subtyping of viral genomes in the examined samples.

**Target**	**Primer and probe sequences**	**Reference**
AIV *M* gene	sep1: AGATGAGTCTTCTAACCGAGGTCG sep2: TGCAAAAACATCTTCAAGTCTCTG sep-probe: FAM-TCAGGCCCCCTCAAAGCCGA-TAMRA	[33]
AIV H5 subtype	H5LH1: ACATATGACTACCCACARTATTCAG H5RH1: AGACCAGCTAYCATGATTGC H5PRO: FAM-TCWACAGTGGCGAGTTCCCTAGCA-TAMRA	[34]
AIV H6 subtype	IAV-H6-1666F: CTTGGTGTGTATCAAATYCTTGC IAV-H6-1776R: CATTGARCCATTTGARCACATCCA IAV-H6-1693: FAM-TATAGTACGGTATCGAGCAGYCT-MGB	[35]
AIV H9 subtype	Forward: GGAAGAATTAATTATTATTGGTCGGTAC Reverse: GCCACCTTTTTCAGTCTGACATT Probe: FAM-AACCAGGCCAGACATTGCGAGTAAGATCC-TAMRA	[36]
AIV N1 subtype	Forward: TAYAACTCAAGGTTTGAGTCTGTYGCTTG Reverse: ATGTTRTTCCTCCAACTCTTGATRGTGTC Probe: FAM-TCAGCRAGTGCYTGCCATGATGGCA-TAMRA	[35]
AIV N2 subtype	Forward: TGGACAGGGAACAACACTAAAC Reverse: ACAAGCCTCCCATCGTAAAT Probe: TXRED-CAAATGAAATGGAACACCCAACTCAT-BHQ23	[35]
AIV N8 subtype	N8-1296F: TCCATGYTTTGGGTTGARATGAT N8-1423R: GCTCCATCRTGCCAYGACCA Probe: FAM-TCHAGYAGCTCCATTGTRATGTGTGGAGT-TAMRA	[35]
IBV	AIBV-fr: ATGCTCAACCTTGTCCCTAGCA AIBV-as: TCAAACTGCGGATCATCACGT AIBV-TM: FAM-TTGGAAGTAGAGTGACGCCCAAACTTCA-TAMRA	[37]
IBDV	F/AUS GU: TCACCGTCCTCAGCTTACCCACATC R/AUS GL: GGATTTGGGATCAGCTCGAAGTTGC	[38]

### Histopathological examination

Representative tissue samples were collected from naturally infected and recently deceased young ostriches suspected of NDV infection. Following necropsy under sterile conditions, specimens from the trachea, lungs, liver, proventriculus, and intestines were immediately fixed in 10% neutral-buffered formalin for 48 h.

After fixation, tissues were processed routinely for paraffin embedding. Samples were dehydrated through ascending grades of ethanol, cleared in xylene, embedded in molten paraffin wax, and sectioned at a thickness of 4–5 μm using a rotary microtome. Tissue sections were mounted on glass slides and stained with H&E. Histopathological examination was performed using a Leica DM 500 light microscope (Leica Microsystems, Wetzlar, Germany) [44].

Histopathological lesions were evaluated using a semiquantitative scoring system. Tissue sections without detectable lesions were assigned a score of 0, whereas slight, moderate, and severe lesions received scores of 1, 2, and 3, respectively [45].

### Biosafety and containment

All procedures involving NDV-positive samples, virus isolation using SPF-ECE, and molecular analyses were conducted in a biosafety level 2 laboratory following institutional biosafety and biosecurity regulations for handling avian avulaviruses. Appropriate personal protective equipment was worn during all laboratory procedures, and infectious materials and biological waste were decontaminated in accordance with established institutional biosafety protocols.

## RESULTS

### Clinical findings, gross lesions, and histopathology

**Figure 2:**
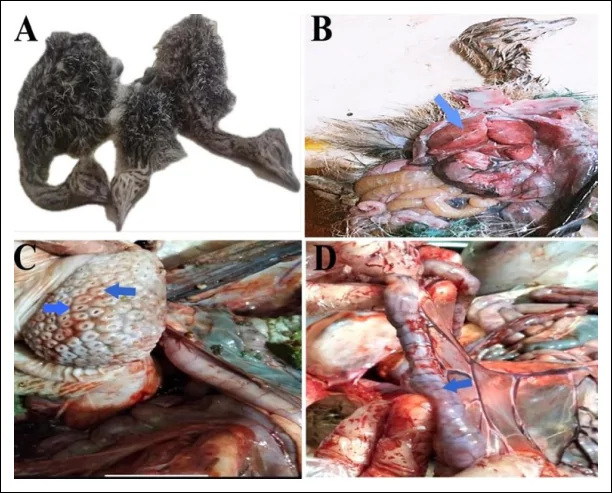
Gross lesions of ostriches naturally infected with Newcastle disease virus. (A) Moribund ostrich showing paralysis of the neck. (B) Hepatomegaly with marked swelling and congestion of the liver (arrow). (C) Glandular papillae of the proventriculus showing prominent swelling and congestion at their tips (arrow). (D) Congestion of the intestinal serosal surface (arrow).

The NDV-positive ostrich farms in the four investigated governorates exhibited mild clinical signs, including depression, anorexia, coughing, nasal and ocular discharge, and sneezing in young birds, with a mortality rate of 4% and a morbidity rate of 30% during the course of the disease (Figure 2A). In a few cases, sudden death occurred without preceding clinical signs, particularly in young or immunocompromised birds.

### Respiratory system histopathology

**Figure 3:**
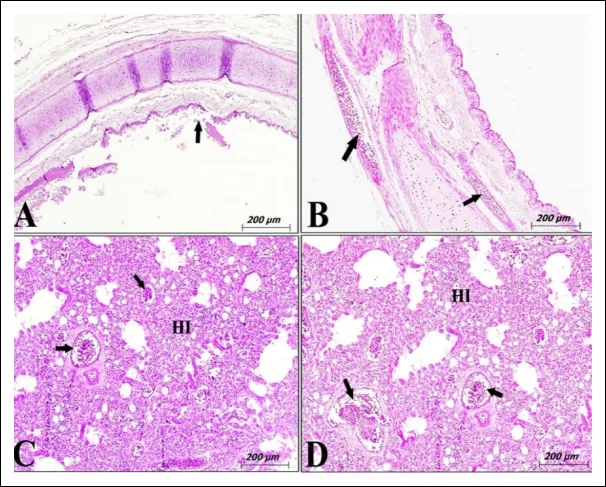
Photomicrographs of respiratory tissues from ostriches naturally infected with Newcastle disease virus. (A) Trachea showing degeneration of the pseudostratified ciliated columnar epithelium with areas of complete epithelial sloughing exposing the basement membrane (arrow). (B) Lamina propria of the trachea showing dense infiltration of lymphocytes and heterophils (arrows). (C and D) Lung showing interstitial pneumonia with marked heterophilic infiltration. The bronchi exhibited epithelial desquamation and necrosis, together with extensive perivascular heterophilic infiltration (arrows). (H&E stain; scale bar = 200 μm).

Gross examination of the trachea of infected ostriches revealed a hyperemic and congested mucosa with the lumen partially occluded by a thick, yellow-to-serosanguineous exudate. Microscopically, these lesions corresponded to degeneration and sloughing of the pseudostratified ciliated columnar epithelium, subepithelial edema, endothelial swelling, and dense infiltration of lymphocytes, plasma cells, and heterophils (Figures 2A, 3A, and 3B).

Meanwhile, the lungs and air sacs appeared grossly heavy, non-collapsed, and diffusely congested, with pulmonary edema, multifocal hemorrhages, fibrinous pleuritis, and air sacculitis. Microscopically, pronounced interstitial pneumonia was observed, characterized by thickened interalveolar septa due to mononuclear cell infiltration, intra-alveolar fibrin deposition and erythrocyte accumulation, bronchial epithelial desquamation, peribronchial lymphoid depletion, and perivascular heterophilic infiltration (Figures 2A, 3C, and 3D).

### Digestive and hepatic pathology

**Figure 4:**
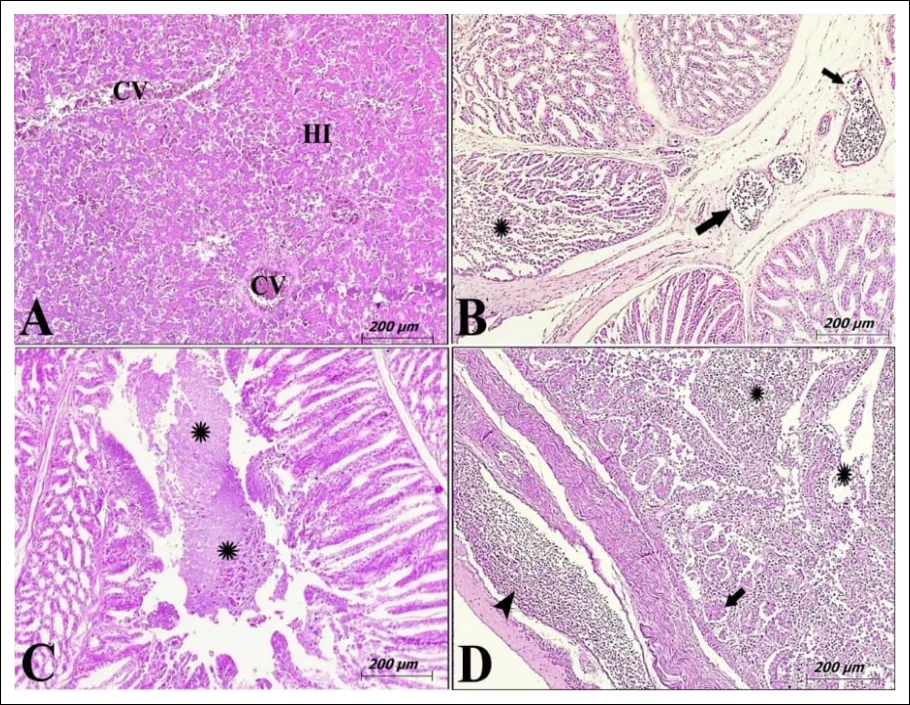
Photomicrographs of different ostrich tissues naturally infected with Newcastle disease virus. (A) Liver showing severe congestion of the central veins (CV) and coagulative necrosis of the hepatic parenchyma (HP). (B) The glandular epithelium of the proventriculus exhibiting degeneration, necrosis (star), and dilated blood vessels within the submucosa (arrows). (C) The overlying mucosa of the proventriculus showing multifocal erosion and ulceration (stars). (D) The intestine showing severe mucosal damage, with the surface epithelium exhibiting erosion or complete necrosis (stars). The lamina muscularis was infiltrated with dense populations of mononuclear cells and heterophils (black arrow). Peyer's patches exhibited marked lymphoid depletion, with extensive areas of necrosis replacing the normal follicular architecture (black arrows). (H&E stain; scale bar = 200 μm).

Gross examination of the proventriculus revealed swollen glandular papillae. Histopathologically, the glandular epithelium exhibited degeneration, necrosis, and loss of normal cellular architecture with luminal eosinophilic debris, accompanied by mucosal ulceration and inflammation of the lamina propria (Figures 2C, 4B, and 4C).

Additionally, gross examination of the intestinal segments (duodenum, ileum, and ceca) revealed mucosal congestion, friability, and fluid, fetid, bile-stained greenish intestinal contents. Microscopically, the villi were shortened, blunted, or fused, with necrosis of the surface epithelium, dilated crypts containing cellular debris, and submucosal hemorrhages (Figures 2D and 4D).

Furthermore, the liver and gallbladder exhibited mild hepatomegaly with rounded borders, a friable consistency, a mottled parenchymal appearance characterized by pale necrotic foci alternating with congested areas, and a bile-distended gallbladder. Histopathological examination revealed disruption of the hepatic architecture due to multifocal-to-coalescing hepatocellular necrosis, sinusoidal dilation, Kupffer cell proliferation, and periportal lymphocytic infiltration (Figures 2B and 4A).

### Lymphoid tissue depletion

Grossly, visceral lymphoid tissues, including Peyer's patches and GALT, were enlarged and congested. Microscopically, marked lymphoid depletion and extensive necrosis replaced the normal follicular architecture of GALT, including Peyer's patches and cecal tonsils, and were accompanied by lymphoid depletion within the proventricular lymphoid nodules (Figures 2D, 4B, 4C, and 4D).

### Histopathological damage scoring

**Figure 5:**
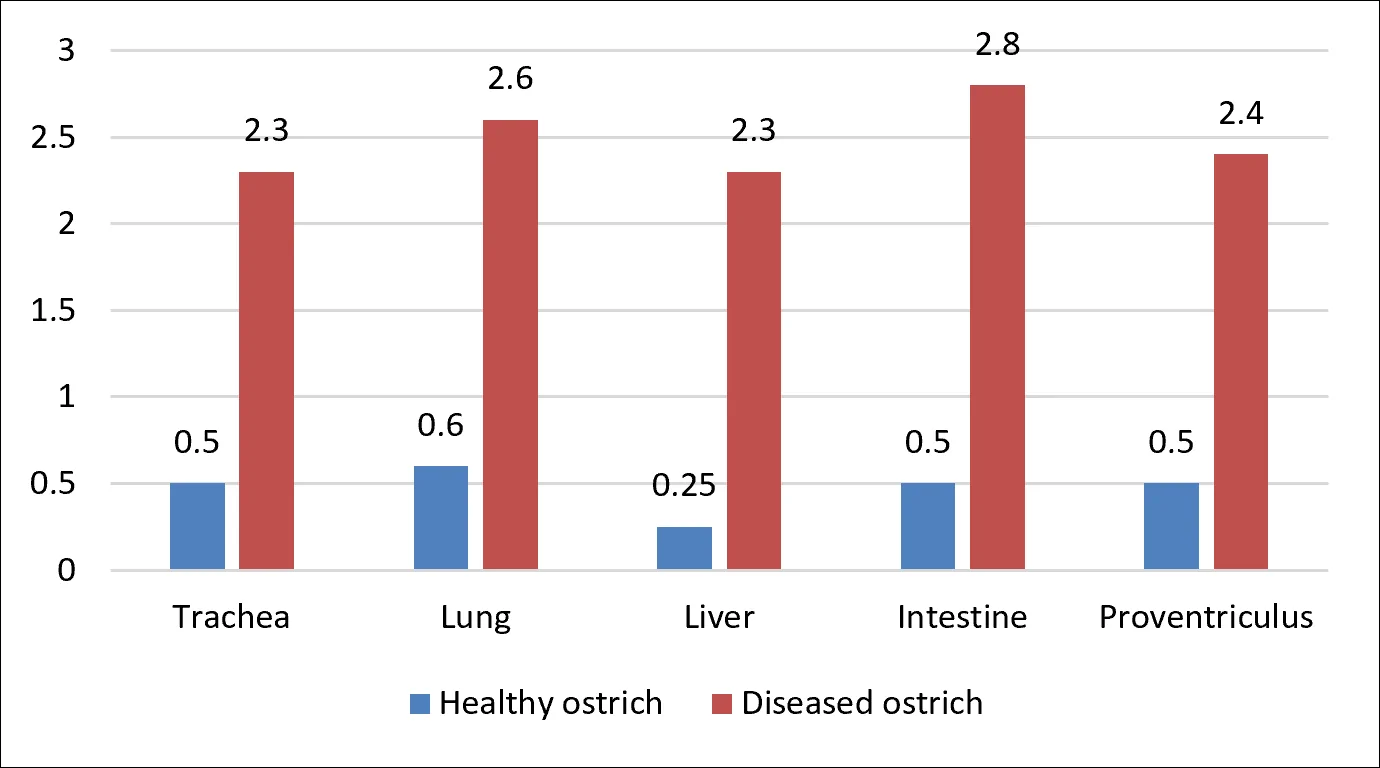
Mean histopathological lesion scores in healthy and diseased ostriches illustrating differences in lesion severity among the examined organs. The intestine was the most severely affected organ in diseased ostriches, followed by the lung.

Semiquantitative lesion scoring of the trachea, lungs, liver, proventriculus, and intestine demonstrated that naturally infected ostriches consistently exhibited markedly higher lesion scores than healthy control birds, indicating severe histopathological damage associated with NDV infection. In contrast, the control group exhibited only minimal lesions, reflecting normal tissue morphology (Figure 5).

### Trachea

The tracheal tissues of the control group had a mean lesion score of 0.5, indicating minimal histopathological alterations and the preservation of normal tissue architecture. Histological examination revealed intact pseudostratified ciliated columnar epithelium with well-preserved cilia and goblet cells.

In contrast, diseased ostriches exhibited a markedly elevated mean lesion score of 2.3, with lesion grades ranging from 2 to 3. These findings were associated with pronounced degenerative and inflammatory changes, including epithelial degeneration, ciliary loss, epithelial desquamation, and inflammatory cell infiltration.

### Lung

Lung tissues obtained from healthy ostriches exhibited a mean lesion score of 0.6, reflecting predominantly normal pulmonary histological architecture. Most of the sections examined displayed clear alveolar spaces, thin interalveolar septa, and patent bronchioles lined by simple columnar epithelium. In contrast, diseased ostriches demonstrated a significantly higher mean lesion score of 2.6, with lesion grades ranging from 2 to 3. Histopathological examination revealed severe disruption of the pulmonary architecture, alveolar deterioration, inflammatory cell infiltration, and vascular alterations.

### Liver

The liver tissues of the control group exhibited a mean lesion score of 0.25, indicating minimal pathological alterations and preservation of normal hepatic architecture. Histologically, hepatocytes appeared polyhedral and were arranged in cords separated by hepatic sinusoids surrounding the central veins. In comparison, diseased ostriches exhibited a significantly higher mean lesion score of 2.3, with lesion grades ranging from 2 to 3. The hepatic lesions were characterized by marked vacuolar degeneration, cytoplasmic alterations, and extensive inflammatory cell aggregates.

### Proventriculus

The proventriculus of healthy ostriches exhibited a mean lesion score of 0.5, reflecting minimal histopathological alterations and preservation of normal tissue organization. Histological sections showed normal mucosal folds and well-developed proventricular glands arranged into lobules separated by delicate connective tissue septa. In contrast, diseased ostriches demonstrated a significantly increased mean lesion score of 2.8, with lesion grades ranging from 2 to 3. Histopathological findings included degeneration and necrosis of the glandular epithelium, dense aggregates of inflammatory cells, and vascular alterations.

### Intestine

Intestinal tissues from healthy ostriches exhibited a mean lesion score of 0.5, indicating minimal histopathological abnormalities and preservation of normal intestinal morphology. Histological examination revealed intact villi, normal crypt architecture, and abundant goblet cells within the mucosa. In contrast, diseased ostriches demonstrated a markedly increased mean lesion score of 2.8, with lesion grades ranging from 2 to 3. The pathological alterations included severe villous blunting and degeneration, crypt distortion, goblet cell depletion, and inflammatory cell infiltration.

### NDV isolation, identification, pathotyping, and coinfection screening

Following three successive passages of NDV in SPF-ECE, the inoculated embryos exhibited mild lesions, including slight congestion of the skin and internal organs. The recovered isolates were HA-positive, with an HA titer of 9 log₂ HA units/mL. Identification of the NDV isolates by the HI assay demonstrated a serum antibody titer of 6 log₂ in all positive samples.

Biological pathotyping confirmed that all NDV isolates belonged to the lentogenic pathotype, with an MDT of 96 h (>90 h indicates a lentogenic strain) and an ICPI of 0.4 (<0.5 indicates a lentogenic strain).

Molecular detection by rRT-PCR targeting the conserved *M* gene confirmed NDV RNA in 8 of the 60 tissue samples collected from eight ostrich farms. Positive samples were detected in farms located in Ismailia (four farms, with the highest prevalence), El-Menofia (two farms), El-Behera (one farm), and El-Sharquia (one farm). The prevalence rates and distribution of positive farms in each governorate are summarized in Table 1.

Furthermore, four representative NDV-positive samples were amplified by conventional RT-PCR for subsequent partial *F* gene sequencing and phylogenetic analysis. Among the eight NDV-positive samples identified by RT-qPCR, four representative isolates were selected for partial *F *gene sequencing based on lower cycle threshold values, higher RNA concentration and purity, and successful amplification by conventional RT-PCR.

All NDV-positive samples were additionally screened for AIV, IBV, and IBDV using specific RT-qPCR assays. No coinfection with these viruses was detected in the ostrich samples examined.

### Sequencing and phylogenetic analysis

**Figure 6:**
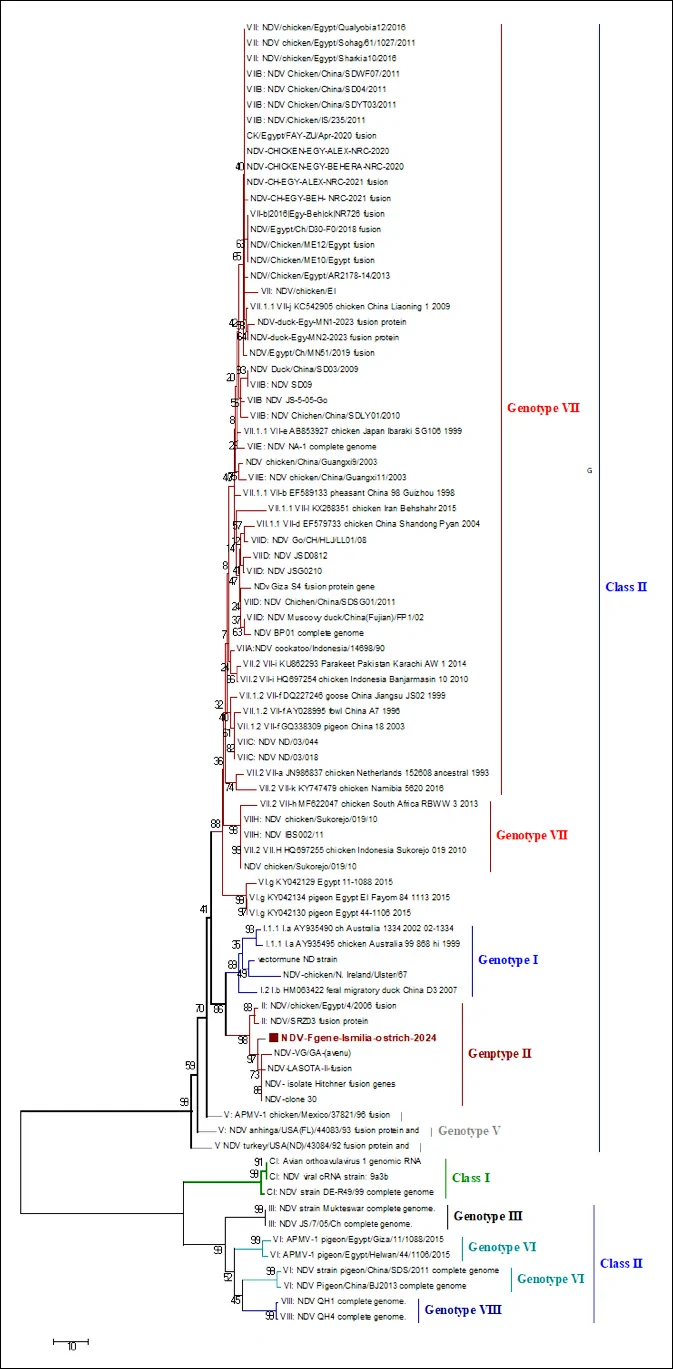
Phylogenetic tree constructed from partial nucleotide sequences of the Newcastle disease virus (NDV) *F* gene together with representative reference strains and Egyptian isolates retrieved from GenBank. The current isolate, NDV-Fgene-Ismailia-ostrich-2024 (GenBank accession no. PX088918), is indicated in the tree. Phylogenetic analysis demonstrated that the ostrich isolate clustered within genotype II, class II, and was closely related to commonly used vaccine strains, including LaSota, Hitchner, Clone 30, and VG/GA. This clustering, together with the lentogenic F protein cleavage-site motif, supports the classification of the isolate as a lentogenic NDV strain. The phylogenetic tree was constructed using the Maximum-Likelihood method with 1,000 bootstrap replicates in MEGA version 7.0.

To unequivocally classify the current ostrich NDV isolate, phylogenetic analysis was performed using the partial *F* gene sequence together with representative reference and vaccine strains available in GenBank (Table 4, Figure 6).

The phylogenetic tree demonstrated that the ostrich NDV isolate, designated NDV-Fgene-Ismailia-ostrich-2024, clustered within genotype II, class II and was assigned GenBank accession number PX088918 (Figure 6).

**Figure 7:**
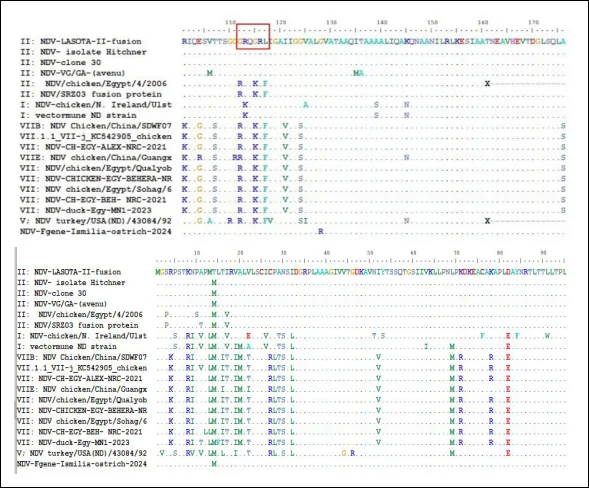
Amino acid sequence alignment of the Newcastle disease virus (NDV) F protein from the ostrich isolate compared with vaccine strains (LaSota, Hitchner, Clone 30, and VG/GA-avenue and representative reference isolates included in the phylogenetic analysis. The F protein cleavage site region (residues 112–117) is highlighted in a red box. The ostrich isolate exhibited the motif ¹¹²GRQGRL¹¹⁷, which is characteristic of lentogenic NDV strains. The upper row indicates the amino acid positions of the LaSota II fusion protein. Amino acid substitutions are represented by letters, identical residues by dots (.), and alignment gaps by dashes (−). The absence of multiple basic amino acids at the cleavage site further supports the molecular classification of the isolate as a low-virulence (lentogenic) NDV strain.

Analysis of the F protein cleavage-site sequence revealed the motif ¹¹²GRQGRL¹¹⁷, which is characteristic of lentogenic NDV strains (Figure 7). In contrast, the polybasic cleavage-site motif ¹¹²RRQKRF¹¹⁷, which is typically associated with velogenic NDV strains, was absent. Therefore, NDV-Fgene-Ismailia-ostrich-2024 was classified as a lentogenic strain based on molecular pathotyping criteria.

Based on the phylogenetic analysis, the current isolate clustered closely with genotype II vaccine strains, including NDV-LaSota-II, NDV isolate Hitchner, NDV-Clone 30, and NDV-VG/GA, as well as genotype II field isolates NDV/chicken/Egypt/4/2006 (Egyptian isolate) and NDV/SRZ03 (Chinese isolate), exhibiting nucleotide identities ranging from 87% to 99% and amino acid identities ranging from 87% to 96%.

In contrast, NDV-Fgene-Ismailia-ostrich-2024 was genetically distinct from the genotype V velogenic strain NDV/turkey/USA(ND)/43084/92, the genotype I vaccine strain Vectormune ND, and the genotype I lentogenic strain NDV/chicken/N. Ireland/Ulster/67, exhibiting nucleotide identities ranging from 74% to 88% and amino acid identities ranging from 75% to 88%.

Similarly, the ostrich NDV isolate exhibited relatively low sequence homology with genotype VII reference strains circulating in China, Namibia, and Egypt, particularly within the hypervariable region of the amplified *F* gene fragment, with nucleotide identities of 80%–82% and amino acid identities of 82%–84% (Table 4).

Collectively, these findings represent the first phylogenetic characterization of a lentogenic genotype II NDV isolate from Egyptian ostriches. The isolate exhibited 97%–99% identity with LaSota-like vaccine strains while remaining genetically distinct from the velogenic genotype VII strains previously reported in ostrich embryos.

Multiple amino acid sequence alignment of the F protein demonstrated the absence of multiple basic amino acid insertions compared with published NDV sequences available in the GenBank database (Figure 7).

Regarding recombination, analysis using RDP5 software with multiple detection algorithms (RDP, BootScan, MaxChi, GENECONV, SiScan, Chimaera, LARD, Phyl-Pro, and 3Seq) revealed no statistically significant recombination events within the partial *F* gene sequence of NDV-Fgene-Ismailia-ostrich-2024. None of the applied methods identified supported recombination breakpoints or parental strains under the selected significance threshold (p > 0.05).

### Novel aspects of the study

This study is the first to integrate virus isolation, comprehensive biological pathotyping (MDT and ICPI), real-time RT-PCR, partial *F* gene sequencing, and systematic histopathological examination of multiple tissues (trachea, lungs, liver, proventriculus, intestines, and other visceral organs) from naturally infected young ostriches. These complementary approaches provide a comprehensive understanding of the biological, molecular, and pathological characteristics of NDV infection in ostriches.

The study provides several novel contributions to the understanding of NDV infection in ratites. First, it presents the first detailed molecular and pathological characterization of a lentogenic genotype II NDV isolated from clinically affected young ostriches in Egypt, in contrast to previous reports that primarily described velogenic genotype VII infection in ostrich embryos. Second, it demonstrates that lentogenic NDV strains, traditionally considered to be of low pathogenicity, can induce significant histopathological lesions in naturally infected young ostriches. Third, the close genetic relatedness (97%–99%) between the identified isolate and commonly used poultry vaccine strains raises important questions regarding possible vaccine spillover and the potential role of ostriches as reservoirs for NDV circulation.

**Table 4:** Nucleotide and amino acid identities of the partially sequenced Newcastle disease virus isolate compared with selected Egyptian isolates, vaccine strains, and representative reference strains.

**Sequence**	**A**	**B**	**C**	**D**	**E**	**F**	**G**	**H**	**I**	**J**	**K**	**L**	**M**	**N**	**O**	**P**	**Q**	**R**	**S**	**T**	**U**
										**											
A	ID	90	98	99	96	98	98	99	98	97	82	82	82	82	84	79	83	75	83	37	82
B	90	ID	89	90	90	89	89	89	89	88	81	81	81	80	84	76	81	74	80	36	80
C	99	90	ID	99	95	100	100	99	100	98	82	82	82	81	84	79	82	74	82	37	81
D	99	90	100	ID	96	99	99	99	99	97	82	82	82	82	84	79	82	75	82	37	82
E	96	90	97	97	ID	95	95	96	95	94	83	83	83	82	84	79	83	74	83	38	82
F	99	90	100	100	97	ID	100	99	100	98	82	82	82	82	84	79	82	75	82	37	82
G	99	90	100	100	97	100	ID	99	100	98	82	82	82	81	84	79	82	74	82	37	81
H	99	90	100	100	97	100	100	ID	99	98	82	82	82	81	84	79	82	75	82	36	81
I	99	90	99	99	96	99	99	99	ID	98	82	82	82	81	84	79	82	74	82	37	81
J	98	89	98	98	95	98	98	98	98	ID	81	81	81	80	83	78	81	73	82	36	80
K	83	82	84	84	84	84	84	84	83	82	ID	99	99	98	88	75	88	87	96	37	98
L	84	82	84	84	84	84	84	84	84	82	99	ID	100	99	89	75	89	88	97	37	99
M	84	82	84	84	84	84	84	84	84	82	99	100	ID	99	89	75	89	88	97	37	99
N	82	81	82	82	82	82	82	82	82	81	98	98	98	ID	88	74	88	87	96	37	98
O	86	85	86	86	86	86	86	86	85	85	89	89	89	88	ID	77	94	79	88	37	88
P	79	76	78	78	78	78	78	78	78	77	75	76	76	74	77	ID	76	82	76	33	74
Q	82	81	81	81	82	81	81	81	81	80	88	88	88	86	94	74	ID	79	88	37	88
R	76	76	76	76	76	76	76	76	76	74	87	88	88	86	78	83	77	ID	89	33	87
S	85	83	85	85	84	85	85	85	84	84	96	97	97	95	88	76	86	90	ID	37	96
T	12	13	12	12	13	12	12	12	12	12	12	12	12	12	13	11	13	9	11	ID	37
U	83	82	84	84	84	84	84	84	83	82	98	98	98	97	88	75	87	87	96	12	ID
							***														

A = VII.1.1 chicken China Liaoning 1 2009 (KC542905); B = VII.2 chicken Namibia 5620 2016 (KY747479); C = VII: NDV-CH-EGY-ALEX-NRC-2021 (MW580389); D = VIIB NDV Chicken China SDWF07/2011 (JQ015295); E = VIIE NDV chicken China Guangxi11/2003 (DQ485231); F = VII NDV/chicken/Egypt/ Qualyobia12/2016 (KY075889); G = VII NDV-CHICKEN-EGY-BEHERA-NRC-2020 (MW580387); H = VII NDV chicken/Egypt/Sohag/61/1027/2011 (MK005980); I = VII NDV-CH-EGY-BEH-NRC-2021 (MW603769); J = VII NDV-duck-Egy-MN1-2023 (PP182340); K = II NDV-LaSota-II (JF950510); L = II NDV isolate Hitchner (AF309418); M = II NDV-Clone 30 (Y18898); N = II NDV-VG/GA (AY562985); O = I Vectormune ND strain (M24692); P = V NDV turkey/USA(ND)/43084/92 (AY741404); Q = I NDV chicken/N. Ireland/Ulster/67 (AY562991); R = II NDV/chicken/Egypt/4/2006 (FJ969395); S = II NDV/SRZ03 fusion protein (DQ097394); T = Class I NDV strain DE-R49/99 (DQ097395); U = NDV-Fgene-Ismailia-ostrich-2024 (PX088918). **: Nucleotide identity % ***: Amino acid identity %. Amino acid and nucleotide identities and divergence of our partially sequenced NDV isolates are comparable to other selected strains, including vaccinal strains. The table presents a comparative alignment of the *F *gene, in which the F nucleotide identity percentage for our isolate ranges from 74% to 99%, comparable to other reference strains. Additionally, the amino acid identity percentages of these isolates range from 75% to 98%, comparable to those of various reference strains. These are the accession numbers: VII.2_VII-k_chicken_Namibia_5620_2016 (KY747479), VII.1.1_VII-j_chicken_China_Liaoning_1_ 2009 (KC542905), VII: NDV-CH-EGY-ALEX- NRC-2021 fusion (MW580389), VIIB: NDV Chicken/China/SDWF07/2011 (JQ015295), VIIE: NDV chicken/ China/Guangxi11/2003 (DQ485231), VII: NDV/chicken/Egypt/Qualyobia12/2016 (KY075889), VII: NDV-CHICKEN-EGY- BEHERA-NRC-2020 (MW580387), VII: NDV chicken/Egypt/Sohag/61/1027/2011 (MK005980), VII: NDV-CH-EGY-BEH- NRC-2021 fusion (MW603769), VII: NDV-duck-Egy- MN1-2023 (PP182340), II:NDV-LASOTA-II (JF950510), II: NDV- isolate Hitchner (AF309418), II:NDV-clone 30 (Y18898),II: NDV-VG/GA-(avenu) (AY562985), I:vectormune ND strain (M24692), V; NDV turkey/USA(ND)/43084/92 (AY741404), I: NDV-chicken/N. Ireland/Ulster/67 (AY562991), II: NDV/chicken/ Egypt/4/2006 (FJ969395), II: NDV/SRZ03 (DQ097394), CI: NDV strain DE-R49/99 (DQ097395).

## DISCUSSION

### Zoonotic relevance and biosafety considerations

NDV is not considered a major zoonotic pathogen; however, occasional cases of mild conjunctivitis and transient influenza-like illness have been reported in individuals exposed to infected birds. Although the zoonotic potential of NDV is very limited, appropriate biosafety practices, personal protective equipment, and proper handling of infected birds and clinical samples should be maintained during outbreak investigations and vaccination procedures [31, 46].

Despite intensive routine vaccination, poultry health remains compromised by a variety of viral diseases, many of which cause substantial mortality and economic losses [47]. In Egypt, numerous NDV outbreaks have been reported in domestic poultry, resulting in significant adverse effects on the poultry industry [48]. The poultry sector plays a major role in the dissemination of NDV among susceptible avian hosts, including ostriches, thereby facilitating disease transmission and environmental contamination. In addition, migratory birds may shed the virus and serve as a potential source of infection for domestic poultry [27, 49].

### Economic importance of ostrich production and NDV impact

Ostrich farming has expanded considerably and is now well established in several countries, including Egypt. In Egypt, ostrich production has become an economically important component of the livestock and poultry sector because of the commercial value of ostrich meat, leather, feathers, and oil. Commercial ostrich farms are concentrated mainly in Ismailia, El-Menofia, El-Behera, Damietta, and El-Sharquia governorates, contributing to food security, rural employment, and export opportunities. Ostrich meat is particularly valued for its high protein content and low cholesterol, making it an attractive alternative to conventional red meat for health-conscious consumers and international markets [50–52].

The economic consequences of NDV infection in ostrich farms extend beyond direct mortality and include reduced growth performance, increased treatment and vaccination costs, impaired reproductive performance, trade restrictions, and reduced market value of ostrich-derived products. Even infection with lentogenic NDV strains may adversely affect meat production, leather quality, feather yield, and export potential. Considering the growing economic importance of ostrich farming in Egypt, continued circulation of NDV may represent an emerging threat to the sustainability and profitability of this industry [53, 54]. Although NDV infection has been reported previously in Egyptian ostrich farms [28, 30], the circulating viruses remain poorly characterized genetically, and only limited information is available regarding their genotypes [29]. Therefore, characterization of NDV isolates circulating among ostrich flocks is essential for improving the understanding of the epidemiology, evolution, and transmission dynamics of the disease. The present study addressed this knowledge gap through molecular characterization of NDV isolates by sequencing and phylogenetic analysis of the *F *gene together with comprehensive pathological evaluation of naturally infected tissues.

### Clinical and gross pathological findings

The clinical signs and gross lesions observed in NDV-infected ostriches were generally consistent with those reported in both experimental and naturally infected ostriches and other avian species. Unlike previous reports describing velogenic genotype VII infection in ostrich embryos, the lentogenic genotype II isolate identified in the present study induced remarkable lesions in the respiratory, digestive, hepatic, and lymphoid tissues. Clinically, affected ostrich flocks from four Egyptian governorates exhibited mild respiratory signs, with an overall mortality rate of 4%. These observations agree with a previous study [55], which reported respiratory and neurological manifestations in experimentally infected ostriches, particularly among young birds, suggesting that disease severity is influenced by age and immune status.

Gross pathological examination revealed lesions involving the trachea, lungs, liver, proventriculus, and intestines that were compatible with infection by lentogenic NDV strains. The principal lesions included mild tracheitis, pulmonary congestion and edema, hepatomegaly with pallor and focal necrosis, and swelling of the proventricular papillae. Similar gross pathological findings have been described previously [56]. Mild hepatomegaly, accompanied by hepatic congestion, observed in the present study is also consistent with the previous findings [57], which reported multifocal hepatic necrosis and congestion in ostriches experimentally infected with NDV. Likewise, intestinal lesions were characterized primarily by mucosal congestion. Disease severity appeared to be age-dependent, with younger birds developing more severe systemic manifestations, as previously reported [29]. Furthermore, although many clinical and pathological findings resemble those observed in chickens and turkeys, ostriches generally develop milder respiratory lesions and more variable intestinal involvement, likely due to species-specific differences in host immune responses and viral replication dynamics [58, 59].

### Histopathological changes and tissue tropism

Microscopic examination demonstrated that NDV infection induced substantial lesions in multiple organs. Degeneration and desquamation of the pseudostratified ciliated columnar epithelium of the trachea, accompanied by subepithelial edema and inflammatory cell infiltration, indicate direct viral injury to the respiratory mucosa. Similar lesions have been reported in chickens infected with lentogenic NDV strains, in which epithelial damage compromises mucociliary clearance and predisposes birds to secondary bacterial infections, including those caused by *Escherichia coli* [60, 61].

Pulmonary lesions consisted predominantly of interstitial pneumonia characterized by thickened interalveolar septa, intra-alveolar exudation, bronchiolar epithelial necrosis, and marked heterophilic infiltration. Comparable pulmonary lesions have been described in ostriches [30] and chickens infected with NDV, reflecting vascular injury and severe inflammatory responses. Peribronchiolar lymphoid depletion further supports the immunosuppressive nature of NDV infection [55].

Hepatic lesions, including sinusoidal lymphocytic infiltration and vascular congestion, were compatible with lesions previously associated with lentogenic NDV strains in several avian species [62]. Similar mild hepatic lesions have also been reported in a previous study [63]. Histopathological alterations observed in the proventriculus, including glandular epithelial degeneration, necrosis, mucosal ulceration, and lymphoid depletion, further demonstrate the affinity of NDV for glandular and lymphoid tissues. The eosinophilic material observed within glandular lumina most likely represented necrotic cellular debris derived from damaged epithelial cells. These lesions have been consistently reported in both natural and experimental NDV infections.

The intestinal lesions, particularly within the ileum and ceca, were characterized by villous atrophy, crypt necrosis, and marked lymphoid depletion. Destruction of GALT, including Peyer's patches and cecal tonsils, emphasizes the immunosuppressive characteristics of NDV infection and explains the increased susceptibility of affected birds to secondary enteric infections [64].

### Virus isolation, biological pathotyping, and molecular detection

Following inoculation into SPF-ECE, infected embryos developed only mild congestion of the skin and internal organs, consistent with previous reports [26, 56, 65]. The recovered isolates were HA-positive, with an HA titer of 9 log₂ HA units/mL and an HI titer of 6 log₂. Biological pathotyping confirmed that all isolates were lentogenic, with an MDT of 96 h and an ICPI of 0.4. These findings agree with previous reports [26, 63, 73–75] but differ from a previous study [28], which described velogenic NDV infection associated with high embryonic mortality in Egyptian ostriches. Similar observations have also been reported elsewhere [13, 69].

Molecular analysis by rRT-PCR confirmed NDV infection in eight samples, of which four representative isolates were subjected to partial *F* gene sequencing. These findings are consistent with previous molecular investigations [26–29, 70]. Importantly, this study documents the circulation of genotype II lentogenic NDV in non-vaccinated commercial ostrich flocks distributed across multiple Egyptian governorates. The detection of genetically related genotype II viruses across geographically separated farms suggests ongoing environmental exposure and potential epidemiological connections among ostrich farms, backyard poultry, and commercial chicken production systems.

### Pathogenicity of lentogenic NDV in young ostriches

Although the identified isolate was classified as lentogenic based on the F protein cleavage-site motif, MDT, and ICPI values, clinically affected ostriches still developed significant pathological lesions. This apparent discrepancy may be explained by several factors, including the high susceptibility of young ostrich chicks, immature immune responses, environmental and management stressors, infectious dose, and possible concurrent bacterial infections. Previous studies demonstrated that young ostriches are considerably more susceptible to NDV infection than adult birds, even following exposure to low-virulence strains [71]. Species-specific host–virus interactions and differences in innate immune responses may also contribute to the observed clinicopathological manifestations. Furthermore, free-range production systems, transportation stress, nutritional deficiencies, and secondary bacterial infections may exacerbate disease severity, allowing lentogenic strains to produce clinically significant disease [72]. Similar observations have recently been reported for lentogenic and vaccine-related NDV strains under field conditions [73].

### Phylogenetic characterization and genotype distribution

Phylogenetic analysis based on the partial *F* gene demonstrated that the ostrich isolate belonged to genotype II, class II and possessed the characteristic lentogenic cleavage-site motif ¹¹²GRQGRL¹¹⁷. These findings agree with a previous report [29], which identified four major NDV genotypes (II, III–IV, VI, and VII) among ostrich isolates, indicating substantial genetic diversity. Similarly, Ren *et al. *[26] identified a lentogenic NDV strain from farmed ostriches in China using whole-genome sequencing, whereas Elboraay *et al. *[30] also reported a lentogenic cleavage-site motif in an ostrich NDV isolate. In contrast, Ghaly *et al. *[28] described a velogenic Egyptian ostrich isolate possessing the cleavage-site motif ¹¹²RRQKRF¹¹⁷. Collectively, these findings suggest that NDV strains circulating in Egyptian ostriches do not originate from a single ancestral lineage.

The present isolate exhibited very high genetic similarity to genotype II vaccine-related strains previously reported in Egyptian poultry, including LaSota-like and Clone 30-like viruses, with nucleotide identities of 98%–99% and amino acid identities of 97%–98%. These findings support previous reports demonstrating the protective efficacy of genotype II vaccines in ostriches [55, 66, 70]. At the same time, they suggest that ostriches may serve as reservoirs or silent carriers of lentogenic NDV strains that facilitate virus transmission between ostriches and domestic poultry.

### Vaccine-related genotype II strains and epidemiological implications

The close phylogenetic relationship between the present isolate and genotype II vaccine strains raises concerns about the silent circulation of vaccine-derived NDV strains in the field. Live-attenuated vaccines, particularly LaSota-like viruses, may spread horizontally among susceptible birds and persist in poultry-dense environments, thereby contributing to viral maintenance and evolution [73, 74]. Although genotype II vaccines are considered safe, prolonged circulation under field conditions could facilitate viral adaptation, accumulation of mutations, or recombination with circulating virulent strains [75, 76].

Additional factors that may facilitate virus dissemination include environmental contamination from vaccine virus shedding, mechanical transmission via personnel, equipment, vehicles, feed, or water, and exposure to wild or feral birds that act as biological or mechanical carriers. Previous studies have suggested that vaccine-derived NDV strains may contribute to viral evolution and, under certain circumstances, may increase in virulence following repeated bird-to-bird transmission [74, 77]. Furthermore, several Egyptian studies have frequently detected genotype II vaccine-related viruses in vaccinated commercial poultry, particularly in broiler and backyard chicken flocks [52, 76, 78]. Collectively, these findings support the hypothesis that genotype II vaccine-related viruses circulate among multiple avian hosts in Egypt, including ostriches.

### Comparison with genotype VII strains and recombination findings

The present isolate exhibited relatively low genetic similarity to genotype VII chicken isolates circulating in Egypt, sharing nucleotide identities of 80%–82% and amino acid identities of 82%–84%. These findings differ from those of Ghaly *et al. *[28], who reported very high similarity (98.9%–99.2%) between Egyptian ostrich isolates and genotype VIIb chicken strains. Moreover, no evidence of recombination was detected within the partial *F* gene sequence, whereas Yin *et al.* [29] previously reported recombination between genotype II and genotype VII NDV isolates obtained from ostriches and chickens.

### Implications for ostrich farming and NDV surveillance

These observations suggest that NDV infection in Egyptian ostriches may originate from multiple epidemiological sources, including infected poultry populations. Because ostriches are commonly raised under open-air, free-range management systems, exposure to infected domestic poultry and wild birds is likely to facilitate the introduction and maintenance of viruses. Considering the high nutritional value, disease resistance, and international commercial importance of ostrich products [79], continuous molecular surveillance, strengthened biosecurity, and routine molecular characterization of circulating NDV strains are essential to minimize the impact of NDV on the expanding ostrich industry and to support effective disease prevention and control strategies.

## CONCLUSION

This study provides the first comprehensive molecular, pathological, and phylogenetic characterization of naturally circulating lentogenic genotype II NDV in young ostriches in Egypt. Molecular analysis identified genotype II, class II NDV carrying the characteristic lentogenic F protein cleavage-site motif (¹¹²GRQGRL¹¹⁷), which was further confirmed by biological pathotyping (MDT = 96 h and ICPI = 0.4). Despite its lentogenic nature, the virus produced evident clinicopathological changes, including respiratory, hepatic, gastrointestinal, and lymphoid lesions, demonstrating that lentogenic NDV strains can induce clinically relevant disease in susceptible young ostriches under field conditions. Phylogenetic analysis revealed that the isolate was closely related to genotype II vaccine-like strains, with 97%–99% sequence identity, yet genetically distinct from the virulent genotype VII strains currently circulating in Egyptian poultry. In addition, no evidence of recombination was detected within the analyzed *F* gene fragment.

These findings have important practical implications for disease surveillance and control in the expanding ostrich industry. The close genetic relationship between the identified isolate and vaccine-related strains highlights the need for continuous molecular surveillance to differentiate field and vaccine-derived viruses, evaluate vaccine performance, and improve biosecurity practices in ostrich production systems. Enhanced monitoring of interactions among ostriches, commercial poultry, backyard flocks, and wild birds is also essential to reduce the risk of virus dissemination.

A major strength of this study is the integration of virus isolation, biological pathotyping, molecular detection, phylogenetic characterization, recombination analysis, and detailed histopathological evaluation, providing a comprehensive understanding of NDV infection in naturally infected ostriches. Nevertheless, the study was limited by the relatively small number of sequenced isolates and the use of partial *F* gene sequencing rather than complete genome analysis. Future studies incorporating whole-genome sequencing, larger sample sizes, longitudinal surveillance, and investigation of vaccine-derived virus transmission dynamics will further clarify the epidemiology and evolution of NDV in ostriches.

Overall, the present findings improve the current understanding of NDV infection in ostriches and provide valuable baseline information for molecular epidemiological surveillance, vaccine evaluation, and the development of effective prevention and control strategies to support the sustainable growth of the ostrich industry in Egypt.

## DATA AVAILABILITY

The data supporting the findings of this study are included within the manuscript. The nucleotide sequence of the NDV isolate generated in this study has been deposited in the National Center for Biotechnology Information (NCBI) GenBank database under accession number PX088918**.**

## AUTHORS’ CONTRIBUTIONS

EAMS: Conceived and designed the study, collected and processed the samples, performed the virological experiments, analyzed the data, drafted the manuscript, and reviewed the final version. AMZ: Performed the phylogenetic analysis and contributed to data interpretation. AF and RME: Conducted the histopathological examinations and interpreted the pathological findings. SA: Participated in the virological laboratory experiments. IE: Participated in virological laboratory experiments, contributed to study supervision, data interpretation, and critical revision of the manuscript. All authors have read and approved the final version of the manuscript.
